# Nesfatin-1 as a Potential Biomarker for Ischemic Stroke: A Case-Controlled Study of a Comparative Analysis of Patients with and Without Internal Carotid Artery Stenosis

**DOI:** 10.3390/diagnostics15060664

**Published:** 2025-03-10

**Authors:** Şennur Delibaş Kati, Serkan Özben, Ertan Küçüksayan, Mert Van, Esra Yeğin Cilli, Aylin Yaman, Tomris Özben

**Affiliations:** 1 Department of Neurology, Antalya Health Research Center, University of Health Sciences, 07100 Antalya, Türkiye; serkanozben@yahoo.com (S.Ö.); mertvan8@gmail.com (M.V.); yaman.aylin@yahoo.com (A.Y.); 2 Department of Biochemistry, Faculty of Medicine, Alanya Alaaddin Keykubat University, 07425 Antalya, Türkiye; ertankucuksayan@gmail.com; 3 Department of Neurology, Antalya Korkuteli State Hospital, 07800 Antalya, Türkiye; esrayegin_@hotmail.com; 4 Department of Medical Biochemistry, Faculty of Medicine, Akdeniz University, 07070 Antalya, Türkiye; ozben@akdeniz.edu.tr

**Keywords:** stroke, biomarker, internal carotid artery, stenosis, nesfatin-1

## Abstract

**Objectives:** Recently, the need for early diagnosis of modifiable risk factors involved in the etiology of stroke has been highlighted in the literature. Nesfatin-1 is a peptide expressed in the central nervous system and peripheral tissues and has been used as a biomarker in recent years. This study aimed to determine the association of ischemic stroke with internal carotid artery stenosis according to nesfatin-1 level and whether it could be used as a biomarker. **Methods:** A total of 118 patients were included in the study. Three groups were defined: acute stroke patients with symptomatic internal carotid artery stenosis, acute stroke patients without internal carotid artery stenosis, and a control group. Nesfatin-1 levels were measured and compared. **Results:** The median value was 22 pg/mL in acute stroke patients with internal carotid artery stenosis, 24.3 pg/mL in acute stroke patients without internal carotid artery stenosis, and 46.4 pg/mL in the control group. There is a difference between the median values of nesfatin-1 according to the stroke groups with the control group (*p* < 0.001). When a cut-off value of ≤30.62 was taken for nesfatin-1, an AUC value of 0.773 indicated statistical significance (*p* < 0.001). Sensitivity was 77.03%, specificity 83.33%, PPV 90.48%, and NPV 63.83%. The main limitations of our study are the small sample size and the fact that the function of nesfatin-1 is not completely known. **Conclusions:** Although we found that nesfatin-1 levels were lower in ischemic stroke patients compared to controls, its diagnostic potential indicates a moderate discriminatory ability with an AUC value of 0.773. Therefore, whether it is suitable for clinical use will be demonstrated by studies in larger and multicenter cohorts.

## 1. Introduction

Ischemic stroke is one of the leading causes of death [1]. Many mechanisms may be interrelated in the etiology of ischemic stroke. The best-known and accepted etiologic causes are atherosclerosis, thrombosis, and embolism [2]. Each or more than one of these causes may play a role in the etiology of stroke. Carotid artery stenosis resulting from atherosclerosis is among the important and modifiable risk factors for stroke [3]. Early diagnosis of a modifiable risk factor in the etiology of a disease with high mortality and morbidity such as stroke is a topic of interest in the current literature as it may provide a significant advantage in treatment planning.

Nesfatin-1 is a peptide with a highly conserved amino acid sequence in mammals, known to be mainly associated with appetite regulation, widely expressed in the central nervous system (CNS) and peripheral tissues, and increasingly used as a biomarker in recent years [4,5,6]. In peripheral structures, the main source of nesfatin-1 in serum is white adipose tissue. Nesfatin-1 has been shown to play a role in blood glucose regulation and regulation of fat storage, which are involved in the etiology of stroke [7,8,9,10]. Nesfatin-1 is widely expressed in the paraventricular nucleus, arcuate nucleus, supraoptic nucleus, and hypothalamic nuclei in the CNS [5,11,12,13]. Studies on the effects of nesfatin-1 on the CNS are much fewer in the literature than studies on its peripheral effects. These studies, both at the clinical and laboratory levels, are related to neuropsychiatric conditions, such as epilepsy, stress, sleep disorders, anxiety, and depression [14,15,16,17]. High levels of nesfatin-1 have been detected in patients with major depressive disorder or epilepsy and low levels of nesfatin-1 in patients with generalized anxiety disorder [18,19,20].

Acute ischemic stroke treatment aims to restore blood flow at an appropriate time. However, increased blood flow causes an inflammatory response in brain tissue and cellular damage may increase with apoptosis resulting from cell necrosis [21,22]. Many studies have shown that serum nesfatin-1 levels are associated with physiological and clinical recovery after cerebral ischemia and are protective against ischemia/reperfusion injury [23,24,25]. There are very few studies in the literature showing the relationship between ischemic stroke and nesfatin-1. In one study, nesfatin-1 concentration was found to be lower in patients with ischemic stroke and this was associated with stroke severity [26]. From this point of view, the relationship between internal carotid artery (ICA) stenosis, among the preventable causes of stroke, and stroke can be considered an interesting issue. Carotid artery stenosis is a pathology in which plaques composed of free fatty acids and fatty cholesterol deposits narrow the carotid artery lumen and reduce blood flow [27]. In the literature, nesfatin-1 has been shown to inhibit FFA-induced endothelial inflammation through its receptor at the molecular level [28]. However, there is no study in the literature evaluating the relationship between nesfatin-1 and carotid artery stenosis, an important etiologic cause in stroke patients, and evaluating whether nesfatin-1 level would be a predictor. To make this evaluation properly, we compare stroke patients with ICA stenosis, stroke patients without ICA stenosis, and a control group.

This study aimed to evaluate the nesfatin-1 levels of ischemic stroke patients with ICA stenosis and ischemic stroke patients without ICA stenosis by comparing them with a control group and to determine the association of ischemic strokes with ICA stenosis according to nesfatin-1 level, and whether it can be used as a biomarker.

## 2. Methods

### 2.1. Patients and Study Design

This study was a prospective clinical investigation conducted in patients with ischemic stroke confirmed by examination findings, computed tomography, and/or magnetic resonance imaging during hospitalization between February 2021 and October 2023. Acute stroke patients with symptomatic ICA stenosis, acute stroke patients without ICA stenosis, and a control group were determined as the study groups. ICA stenosis was evaluated using the North American Symptomatic Carotid Endarterectomy Trial (NASCET) classification, with ICA stenosis confirmation by CT or MR angiography in stenoses greater than 50%. Exclusion criteria were high blood pressure (systolic 220 mm Hg/diastolic 140 mm Hg), decompensated heart failure and history of acute myocardial ischemia (<6 months), uncontrolled diabetes mellitus, and peripheral vascular disease. Patients receiving intravenous tissue plasminogen activator or endovascular therapy for acute stroke were excluded because these treatment options may affect the duration of ischemia-reperfusion injury and consequently affect nesfatin-1 levels. In addition, patients with hemorrhagic stroke and secondary causes of stroke etiology (stroke due to all type of malignancy, bleeding diathesis., traumatic causes, etc.) were also excluded. According to these criteria, 118 patients were included in the study.

Clinical information and demographic data were obtained from the patients’ medical records. Standard stroke treatment protocols were applied to all patients. Ischemic stroke subtypes were divided into 4 groups by Trial of Org 10172 in Acute Stroke Treatment (TOAST) criteria. These were classified as large artery atherosclerosis, cardiac embolism, small artery occlusion, idiopathic, and others [28]. Patients were evaluated by a stroke neurologist upon admission and stroke severity was assessed using the National Institutes of Health Stroke Scale (NIHSS). Patients were functionally assessed using the modified Rankin Scale (mRS). A good functional outcome was defined as an mRS of 0–2 points, while a poor outcome was defined as an mRS of 3–6 points [29]. Written informed consent was obtained from all participants in the study and the approval of the Ethics Committee (ethics committee decision 2021-226 and decision number 13/9) of our hospital was obtained. In addition, the study was planned according to the protocol by the ethical rules of the 1975 Declaration of Helsinki.

### 2.2. Blood Collection and Human Nesfatin-1 Quantification

Blood samples were collected venously after the diagnosis of stroke was confirmed within 12 h (ischemia-reperfusion-induced inflammation and oxidative stress affect the blood–brain barrier in 12 h) following examinations performed in the emergency department [30]. Blood samples were stored at −80 °C until the number planned for the study was reached. Once the planned number was reached, the samples were thawed only once before use. Human nesfatin-1 levels were measured using enzyme-linked immunosorbent assay (ELISA) kits (SunLong Biotech Co., LTD, Hangzhou, China; catalog number: SL2458Hu). According to the instructions, sensitivity, assay range, and the intra-assay and interassay coefficients of variation are 1.5 pg/mL, 8 pg/mL–400 pg/mL, <10%, and <12% for Nes-1, respectively.

### 2.3. Statistical Methods

The data were analyzed with IBM SPSS V23. The results are expressed as percentages for categorical variables and mean ± standard errors for continuous variables. Compliance with normal distribution was examined by the Shapiro–Wilk test. Mann–Whitney U and Kruskal–Wallis tests were used to compare nonnormally distributed continuous data between groups. Spearman’s rho correlation coefficient was used to evaluate the relationships between quantitative data. Receiver operating characteristic (ROC) analysis was used to determine the appropriate cut-off value for nesfatin-1. Categorical data were analyzed using Fisher’s exact test. A significance level of *p* < 0.05 was determined. The sample size was calculated using the G*Power V. 3.1.9.6 program. With 95% confidence (1 − α), 95% test power (1 − β), and f = 0.353 effect size (in the ANOVA test, the effect size was calculated using Cohen’s f coefficient.), the number of cases that should be included in a study of 3 groups was determined as 114 in total. Since the study was completed with 118 cases, the power of the test was found to be 95.2%, according to the post hoc power analysis.

## 3. Results

A total of 118 patients were included in the study and observed. These patients were divided into three groups. In all groups, 32 (27.2%) of the patients were female. Median age values differed according to the group (*p* < 0.001). The median age was 71.07 + 8.90 years in the acute stroke group with ICA stenosis, 68.94 + 14.58 years in the acute stroke group without ICA stenosis, and 63.37 ± 9.56 years in the stroke-free group. The median age of cases in the control group differed from the others. The distribution of diabetes mellitus, chronic heart disease, hyperlipidemia, and renal failure was similar in all three groups. Other characteristic data about TOAST criteria and mRS results are detailed in Table 1.

The median values of left atrium (LA) diameter differed between groups (*p* = 0.009). The median value was 41 mm in acute stroke patients with ICA stenosis, 40 mm in acute stroke patients without ICA stenosis, and 35 mm in the control group. The difference in LA diameter was due to acute stroke patients with ICA stenosis and chronic stroke patients. The median value of hemoglobin (Hgb) was different between groups (*p* = 0.002). The median value was 12.3 g/dL in acute stroke patients with ICA stenosis, 13.7 g/dL in acute stroke patients without ICA stenosis, and 14 g/dL in the control group. The difference in Hgb is due to acute stroke patients with ICA stenosis and chronic stroke patients. Acute stroke patients without ICA stenosis did not differ from the other groups. Mean platelet volume (MPV) median values differed between groups (*p* = 0.002). The median value was 10.7 fl in acute stroke patients with ICA stenosis, 10.8 fl in acute stroke patients without ICA stenosis, and 9.9 fl in the control group. The value obtained in the control group differed from the groups with and without ICA stenosis. C-reactive protein (CRP) median values differed between groups (*p* = 0.015). The median value was 8.6 in acute stroke patients with ICA stenosis, 8.7 in acute stroke patients without ICA stenosis, and 4.1 in chronic stroke patients. The value obtained in the control group differed from the groups with and without ICA stenosis. Calcium median values differed between groups (*p* = 0.002). The median value was 9 in acute stroke patients with ICA stenosis, 9 in acute stroke patients without ICA stenosis, and 9.6 in chronic stroke patients. The value obtained in the control group differs from the group with ICA stenosis. Other parameters did not differ between groups (*p* > 0.05) (Table 2).

There is a significant negative correlation between nesfatin-1 and Hgb in the group with ICA stenosis (r = −0.386; *p* = 0.014). There is also a positive significant correlation between nesfatin-1 and international normalized ratio (INR) and between nesfatin-1 and age (r value = 0.589 and *p* = 0.001). In the group without ICA stenosis, there is a positive significant correlation between nesfatin-1 and D-Dimer (r = 0.448; *p* = 0.017) (Table 3). In addition, there is a difference between the median values of nesfatin-1 according to the group (*p* < 0.001) (Table 4).

**Figure 1:**
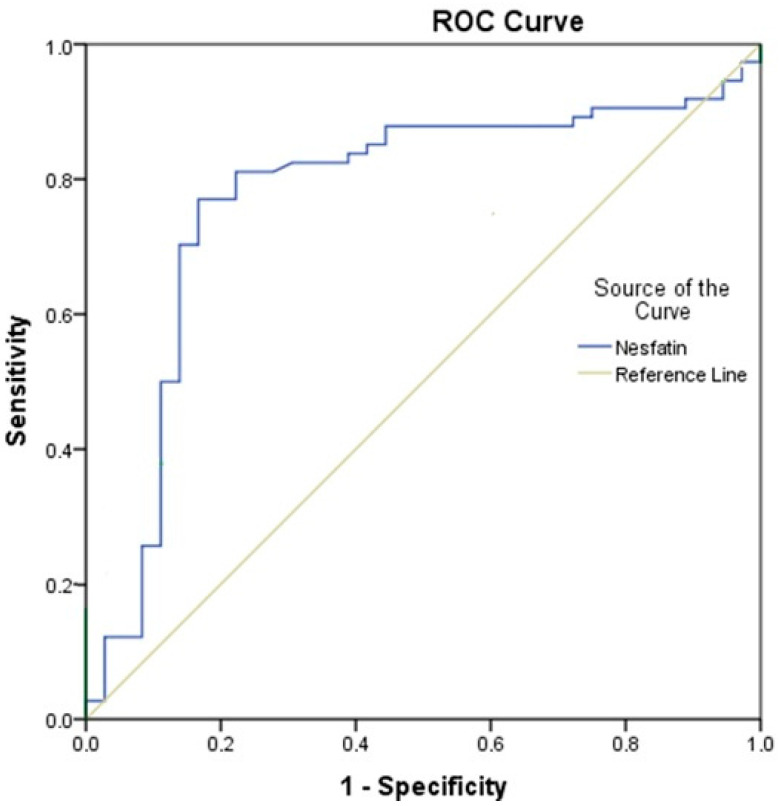
ROC analysis results between acute stroke groups and control group.

When a cut-off value of ≤30.62 was determined for nesfatin-1, the AUC value of 0.773 was considered to indicate statistical significance (*p* < 0.001). Sensitivity was 77.03%, specificity 83.33%, PPV 90.48%, and NPV 63.83% (Table 5) (Figure 1).

## 4. Discussion

Our study showed that serum nesfatin-1 levels in stroke patients were lower than in the control group. There was no significant difference between the nesfatin-1 levels of stroke patients with and without ICA stenosis. In addition, a negative correlation was found between nesfatin-1 and Hgb and a positive correlation was found between nesfatin-1 and INR and age in the group with ICA stenosis. In the group without ICA stenosis, there was a positive correlation between nesfatin-1 and D-Dimer. According to these results, nesfatin-1 levels in acute stroke patients were found to be significantly lower than nesfatin-1 levels in healthy people. In addition, no significant difference was observed in nesfatin levels between patients with and without ICA stenosis when evaluated according to etiological causes and mRS results.

Biomarkers [low-density lipoprotein–cholesterol, glial fibrillary acidic protein (GFAP), lipoprotein-associated phospholipase A2, antibodies against NR2A/NR2B subunits of the N-Methyl-D-Aspartate (NMDA) receptor, neuron-specific enolase, myelin basic protein, heart-type fatty acid-binding protein—HFABP, Parkinson’s disease protein 7—PARK7, and nucleoside diphosphate kinase A—NDKA] that have the potential to distinguish stroke from diseases mimicking stroke or from healthy controls have been proposed in many studies aiming to measure their levels in the blood [31,32,33,34]. However, many of these biomarkers have not entered daily clinical practice. Possible reasons for the lack of practical use of biomarkers include the lack of acceptable sensitivity and specificity of these biomarkers and the fact that stroke is a heterogeneous disease with variability in infarct size, location, and cause. In addition, many biomarkers associated with ischemic stroke are not specific to the disease and changes in plasma levels are expected in conditions that cause acute brain injury, such as intracerebral hemorrhage, subarachnoid hemorrhage, and traumatic brain injury.

Nesfatin-1 is a peptide that is associated with appetite regulation and has been proven to be expressed in the CNS and peripheral tissues [4]. In peripheral tissues, it has been shown to play a role in the regulation of gastrointestinal and cardiovascular function and malignancy [5,35,36,37]. In the CNS, it is mostly found in the hypothalamic nucleus and is known to be associated with major depressants, epilepsy, and anxiety. However, there are almost no studies in the literature evaluating nesfatin-1 and ischemic stroke and stroke etiology-related conditions.

In a study evaluating blood concentration of novel adipocytokines in patients with ischemic stroke, the use of proteins, such as omentin-1, irisin, C1q/TNF-related protein-1 (CTRP1), vaspin, and nesfatin-1 as biomarkers in ischemic stroke was investigated. Accordingly, high blood concentrations of omentin-1 and CTRP1 and low blood concentrations of nesfatin-1 and irisin significantly increase the probability of being included in the ischemic patient group [20].

In a review investigating the use of nesfatin-1 as a biomarker and/or potential therapeutic target in neurological diseases, nesfatin-1 levels were shown to be reduced in patients with ischemic stroke. Therefore, it was suggested that it could be used as a biomarker of the severity and prognosis of cerebral ischemia. Furthermore, it has been suggested that exogenous nesfatin-1 supplementation or enhancement of its expression may be an option in treatment [28,38]. In our study, a similar result was obtained and higher nesfatin-1 levels were found in the control group compared to both acute ischemic stroke groups. In a study evaluating low nesfatin-1 levels in acute myocardial infarction, low nesfatin-1 levels were associated with increased sympathetic activity contrary to what is expected in an acute inflammatory process [39]. On the other hand, high nesfatin-1 levels have been measured in diseases involving neurostructural tissues, such as subarachnoid hemorrhage, Alzheimer’s disease and epilepsy [38]. The similar nesfatin-1 levels between stroke groups in our study may be explained by the decrease in blood levels of nesfatin-1 in acute processes such as acute myocardial infarction. The inflammatory process occurring at the time of ischemia may indicate a rapid response decrease in nesfatin-1 levels. Although there are significant etiologic differences between the two groups according to the TOAST classification, the acute effects of a stroke event are thought to primarily affect nesfatin-1 levels. This is also supported by the fact that nesfatin-1 levels do not decrease in chronic diseases such as Alzheimer’s disease and epilepsy. At this stage, the mechanisms related to nesfatin-1 are still unclear, and therefore, studies are needed to show the variability of nesfatin-1 in neurostructural diseases in order to use it as a biomarker.

In our study, three blood parameters were observed to be correlated with nesfatin-1 in the groups with and without ICA stenosis. There is a significant negative correlation between nesfatin-1 and Hgb in the group with ICA stenosis. We suggest that this may be explained by anemia of chronic disease, which may occur in patients with ICA stenosis due to atherosclerosis and associated chronic inflammation. There is also a positive significant correlation between nesfatin-1 and international normalized ratio (INR) and between nesfatin-1 and ICA stenosis. We think that the positive correlation between nesfatin-1 and INR in the group with carotid stenosis may be related to the increase in INR due to the effect of coumadin used by patients with symptomatic ICA stenosis due to cardiac and/or neurologic disease. Finally, there was a significant positive correlation between nesfatin-1 and D-dimer in the group without ICA stenosis. We think that this may be explained by the fact that stroke develops on the basis of embolic etiology and D-dimer, a fibrin degradation product, and increases in patients without stenosis.

When analyzed by group, the nesfatin-1 levels in the control group were almost 2-fold higher than the nesfatin-1 levels of acute stroke patients with or without ICA stenosis. At this stage, we aimed to determine a cut-off value to be used as a biomarker for nesfatin-1. For this purpose, ROC curve analysis was used to examine the ability of serum nesfatin-1 levels to distinguish acute ischemic stroke patients from healthy individuals. According to the results of the analysis, an AUC value of 0.773 was statistically significant when the nesfatin-1 value was ≤30.62 pg/mL (*p* = 0.001). However, although statistically significant, an AUC value of 0.773 indicates that nesfatin-1 is not sufficient to be a fully reliable diagnostic biomarker. Although the group with ICA stenosis had the lowest nesfatin-1 levels, it was not significantly different from the group without ICA stenosis. In conclusion, although it cannot be associated with the presence of ICA stenosis, in this study, we suggest that a nesfatin-1 level below 30 pg/mL in acute stroke could be a parameter supporting a diagnosis of stroke. Nevertheless, we also think that large-scale multi-center studies are needed to determine its true clinical utility.

This study has several limitations. The first limitation is that our study is a single-center study and the sample size is limited. In addition, despite the power calculation, the sample size may be insufficient to detect subtle differences between subgroups. Another limitation of the study is the potential influence of confounding variables. Differences in baseline characteristics, such as age, sex, and pre-existing comorbidities, may have impacted nesfatin-1 levels. Additionally, stroke severity, as measured by NIHSS scores, may contribute to variations in biomarker levels across groups. These confounders were not fully controlled, limiting the ability to draw definite conclusions. The results of the study, and the small number of these and similar studies in the literature, indicate that the study should be conducted with larger samples. In addition, although many studies have been conducted on nesfatin-1, the lack of sufficient information about its function in the CNS and its role in stroke is another important limitation.

## 5. Conclusions

Early identification of risk factors for ischemic stroke not only reduces morbidity and mortality from stroke but also improves quality of life and reduces treatment costs. In our study, we showed that nesfatin-1 was significantly decreased in ischemic stroke patients. However, articles comparing our results are almost impossible to find in the literature. It is very necessary and important to determine biomarkers to be used in the early diagnosis of ischemic stroke for both nesfatin-1 and other molecules.

The findings of this study demonstrate that serum nesfatin-1 levels are significantly lower in acute ischemic stroke patients compared to healthy controls, suggesting its potential as a biomarker for ischemic stroke. However, these results should be interpreted with caution due to the limitations of the study’s design and sample size. While nesfatin-1 shows promise as a potential biomarker in ischemic stroke, the findings of this study require validation in larger, multi-center cohorts with longitudinal designs. Future research should also aim to elucidate the mechanisms underlying nesfatin-1’s role in stroke pathophysiology and evaluate its clinical utility in differentiating stroke subtypes and predicting outcomes.

## Figures and Tables

**Table 1:** Patients’ demographic data according to group: stroke with ICA stenosis, stroke without ICA stenosis, and control.

	Stroke with ICA Stenosis *n*: 40	Stroke Without ICA Stenosis *n*: 34	Control Group *n*: 44	*p* *
Age	71.07 ± 8.90	68.94 ± 14.58	63.37 ± 9.56	
Sex				
Male	31 (77.5)	23 (67.6)	32 (72.8)	0.602
Female	9 (22.5)	11 (32.4)	12 (27.2)	
Hypertension				
(−)	11 (27.5)	12 (35.3)	39 (88.6)	<0.001
(+)	29 (72.5)	22 (64.7)	5 (11.4)	
Diabetes mellitus				
(–)	23 (57.5)	17 (50)	41 (93.2)	<0.001
(+)	17 (42.5)	17 (50)	3 (6.8)	
Chronic heart disease				
(–)	16 (40)	17 (50)	43 (97.7)	<0.001
(+)	24 (60)	17 (50)	1 (2.3)	
Hyperlipidemia				
(–)	20 (50)	14 (41.2)	44 (100)	<0.001
(+)	20 (50)	20 (58.8)	0 (0)	
Renal failure				
(+)	1 (2.5)	0 (0)	0 (0)	---
(–)	39 (97.5)	34 (100)	44 (100)	
TOAST classification				
Large artery	36 (90)	4 (11.8)	---	<0.001
Cardioembolism	3 (7.5)	9 (26.5)	---	
Small-vessel occlusion	0 (0)	6 (17.6)	---	
Undetermined etiology	0 (0)	15 (44.1)	---	
Others	1 (2.5)	0 (0)	---	
mRS			---	
Good (0–2)	32 (80)	29 (85.3)	---	0.859
Bad (3–6)	8 (20)	5 (14.7)	---	

Abbreviations: TOAST: Trial of Org 10172 in Acute Stroke Treatment. * Fisher’s exact test.

**Table 2:** Baseline characteristics among ischemic stroke patients according to group: stroke with ICA stenosis, stroke without ICA stenosis, and control.

	ICA Stenosis (+) Stroke		ICA Stenosis (−) Stroke		Control Group		
	*n*	Mean ± S. Deviation Median (Min–Max)	*n*	Mean ± S. Deviation Median (Min–Max)	*n*	Mean ± S. Deviation Median (Min–Max)	*p*
Stroke time	40	1.2 ± 0.5/1 (1–3)	34	1.2 ± 0.4/1 (1–2)		--	0.823 ^y^
EF	39	54.7 ± 13.3/60 (20–65)	31	58.9 ± 7.6/60 (25–65)	33	60 ± 3.5/60 (50–65)	0.508 ^w^
LA diameter	39	40.9 ± 6.1/41 (30–57)	31	40.4 ± 6.8/40 (30–63)	33	36.6 ± 5.4/35 (24–48)	**0.009** ^w^
NIHSS	40	7.5 ± 6.8/6 (0–24)	34	7.9 ± 6.9/5 (0–25)		--	0.744 ^y^
mRs	40	1.2 ± 1.6/1 (0–5)	34	1.1 ± 1.2/1 (0–4)	36	1 ± 1.2/1 (0–4)	0.940 ^w^
Hb	40	12.2 ± 2.4/12.3 (2.9–16.6)	34	13.4 ± 1.8/13.7 (9.2–16.2)	36	13.8 ± 1.9/14 (7.1–16.7)	**0.002** ^w^
PLT	40	250.2 ± 64.8/249.5 (127–432)	34	241.9 ± 85.8/232.5 (101–475)	36	271.2 ± 113/246.5 (113–746)	0.526 ^w^
RDW	40	15.4 ± 5.3/13.5 (11.9–37.7)	34	13.9 ± 2.3/13.2 (12.2–24.3)	36	14 ± 1.9/13.6 (12.2–23)	0.412 ^w^
MPV	40	16.9 ± 22.2/10.7 (8.9–103)	34	21.5 ± 62.8/10.8 (9–377)	36	9.9 ± 1.3/9.9 (6.1–12.5)	**0.002** ^w^
GFR	40	66.7 ± 22.4/65.5 (17–107)	34	74.6 ± 21.2/75 (33–117)	29	79.1 ± 16.5/78 (49–113)	0.080 ^w^
Cr Cl	40	1.2 ± 0.5/1 (0.7–3.1)	34	1 ± 0.3/0.9 (0.6–1.7)	36	0.9 ± 0.2/0.9 (0.6–1.5)	0.090 ^w^
Ca^++^	35	9.1 ± 0.6/9 (7.2–10.1)	15	9.1 ± 0.6/9 (8.2–10.3)	35	9.5 ± 0.5/9.6 (7.8–10.2)	**0.002** ^w^
HDL	39	41.2 ± 8.2/41 (26–59)	33	43.3 ± 9.8/41 (20–69)	36	47 ± 12.2/45 (30–70)	0.216 ^w^
LDL	39	110.6 ± 38.5/107 (38–196)	33	112.2 ± 33.9/110 (17–202)	36	116.9 ± 34.1/118.5 (56–203)	0.722 ^w^
TG	39	124.3 ± 64.2/112 (55–424)	33	125.8 ± 62/109 (38–238)	35	152.7 ± 90.8/122 (52–511)	0.261 ^w^
TC	39	174.5 ± 41.9/168 (101–263)	33	180.7 ± 46.3/174 (49–277)	36	192.1 ± 38.4/191 (137–300)	0.186 ^w^
Fibrinogen	35	358.9 ± 114.9/355 (67–627)	28	352.3 ± 128.3/348 (150–722)	17	321.9 ± 91.7/311 (199–481)	0.427 ^w^
Ddimer	35	632.6 ± 725.2/317 (96–2759)	28	1503.9 ± 5375.8/317.5 (83–28,836)	17	317.1 ± 288.2/229 (10–934)	0.116 ^w^
INR	40	1.5 ± 1/1.1 (0.9–2.7)	33	1.2 ± 0.4/1.1 (0.9–2.8)	24	1.1 ± 0.1/1 (0.9–1.3)	0.052 ^w^
aPTT	38	31.5 ± 6/31.2 (20–47.6)	32	30.6 ± 6/30 (11.8–48.5)	21	28.8 ± 3.8/29.1 (20–36.9)	0.256 ^w^
Hba1c	39	7.1 ± 2/6.3 (4.4–13.5)	33	6.7 ± 1.6/5.9 (4.7–11.3)	35	6.9 ± 1.9/6.1 (4.8–12.4)	0.445 ^w^
FBS	40	147.1 ± 62.9/130.5 (59–293)	34	148.6 ± 74.9/124 (70–394)	36	128.7 ± 44.4/117 (79–317)	0.602 ^w^
TSH	38	1.9 ± 3.7/1.1 (0–21.6)	33	1.5 ± 1.3/1.1 (0–5.7)	36	1.8 ± 1.3/1.5 (0–6.2)	0.158 ^w^
B12	38	340.5 ± 309.2/213 (49–1526)	33	239.1 ± 183.8/191 (23–891)	36	255.8 ± 215.1/179.5 (61–1001)	0.379 ^w^
Folate	33	9.5 ± 4.6/8.1 (3.5–24.3)	21	10.6 ± 12.4/7.2 (3.9–63.1)	33	8.7 ± 3.6/8.5 (2.1–17.7)	0.674 ^w^
Fe^++^	33	61.3 ± 35.8/55 (13–177)	23	50.4 ± 18.8/45 (24–92)	29	75.2 ± 41.9/76 (12–196)	0.054 ^w^
Ferritin	38	109.3 ± 165.3/55 (9–844)	32	84.7 ± 52.1/68 (22–265)	35	62.7 ± 65.5/42 (2–316)	0.051 ^w^
Sedimentation	37	19.6 ± 17.6/14.4 (2–77)	29	17.4 ± 18.7/11 (2–80)	36	12.6 ± 9.4/10 (2–34)	0.148 ^w^
CRP	38	27.5 ± 43/8.6 (0.5–199.9)	34	20.7 ± 28.1/8.7 (1.2–115.6)	36	6.6 ± 6.5/4.1 (0.2–26)	**0.015** ^w^
Procalcitonin	13	0.2 ± 0.3/0.1 (0–1.2)	24	0.1 ± 0/0.1 (0–0.2)			0.403 ^y^

Abbreviations: EF: ejection fraction, LA: left atrium, NIHSS: National Institutes of Health Stroke Scale, mRS: modified Rankin Scale, Hb: hemoglobin, PLT: platelet, RDW: red cell distribution width, MPV: mean platelet volume, GFR: glomerular filtration rate, Cr Cl: creatinine clearance, Ca^++^: calcium, HDL: high-density lipoprotein, LDL: low-density lipoprotein, TC: total cholesterol, TG: triglyceride, INR: international normalized ratio, aPTT: activated partial thromboplastin time, HbA1c: hemoglobin A1c, FBS: fasting blood sugar, TSH: thyroid-stimulating hormone, B12: vitamin B12, Fe^++^: iron, CRP: C-reactive protein. (^y^ Mann–Whitney U test; ^w^ Kruskal–Wallis test).

**Table 3:** Correlation analysis results (cases with and without ICA stenosis).

	ICA Stenosis (+)			ICA Stenosis(−)		
	Nesfatin			Nesfatin		
	r	*p*	n	r	*p*	N
Stroke time	0.216	0.181	40	0.211	0.230	34
EF	−0.017	0.916	39	0.054	0.771	31
LA diameter	−0.048	0.772	39	−0.004	0.983	31
NIHSS	0.306	0.055	40	0.248	0.157	34
mRS	−0.075	0.644	40	0.173	0.328	34
Hb	**−0.386**	**0.014**	40	−0.229	0.193	34
PLT	0.089	0.586	40	0.098	0.583	34
RDW	0.305	0.056	40	0.099	0.579	34
MPV	−0.069	0.673	40	0.112	0.527	34
GFR	−0.145	0.371	40	−0.181	0.305	34
Cr Cl	−0.012	0.942	40	0.098	0.580	34
Ca^++^	−0.233	0.177	35	−0.304	0.271	15
HDL	0.016	0.924	39	0.044	0.808	33
LDL	0.128	0.438	39	0.181	0.313	33
TG	−0.172	0.296	39	−0.061	0.737	33
TC	0.088	0.596	39	0.108	0.549	33
Fibrinojen	0.015	0.934	35	0.269	0.167	28
D-Dimer	0.316	0.064	35	**0.448**	**0.017**	28
INR	**0.589**	**<0.001**	40	−0.043	0.813	33
aPTT	0.033	0.845	38	−0.248	0.172	32
HbA1c	0.086	0.603	39	0.036	0.841	33
FBS	0.224	0.164	40	0.167	0.344	34
TSH	−0.076	0.648	38	0.185	0.303	33
B12	−0.091	0.586	38	0.005	0.979	33
Folate	−0.285	0.107	33	0.165	0.475	21
Fe^++^	0.148	0.412	33	−0.227	0.298	23
Ferritin	0.097	0.563	38	0.164	0.371	32
Sedimentation	−0.218	0.194	37	0.336	0.075	29
CRP	−0.045	0.790	38	0.160	0.367	34
Procalcitonin	−0.413	0.160	13	0.015	0.944	24

Abbreviations: EF: ejection fraction, LA: left atrium, NIHSS: National Institutes of Health Stroke Scale, mRS: modified Rankin Scale, Hb: hemoglobin, PLT: platelet, RDW: red cell distribution width, MPV: mean platelet volume, GFR: glomerular filtration rate, Cr Cl: creatinine clearance, Ca^++^: calcium, HDL: high-density lipoprotein, LDL: low-density lipoprotein, TC: total cholesterol, TG: triglyceride, INR: international normalized ratio, aPTT: activated partial thromboplastin time, HbA1c: hemoglobin A1c, FBS: fasting blood sugar, TSH: thyroid-stimulating hormone, B12: vitamin B12, Fe^++^: iron, CRP: C-reactive protein.

**Table 4:** Nesfatin values according to group.

	**Nesfatin**	
	***n***	**Mean ± S. Deviation/Median (Min–Max)**
Stroke with ICA stenosis	40	25.7 ± 12.5/22 (11–70) ^a^
Stroke without ICA stenosis	34	40 ± 52.3/24.3 (13.6–258.3) ^a^
Control group	44	97.9 ± 107.7/46.4 (33.6–445.9) ^b^
*p* *		<0.001

* Kruskal–Wallis test; ^a,b^ There is no difference between groups with the same letter in each group. There is a difference between the median values of nesfatin according to group (*p* < 0.001). The median value was 22 in acute stroke patients with ICA stenosis, 24.3 in acute stroke patients without ICA stenosis, and 46.4 in the stroke-free group. While there was no difference between the groups with and without ICA stenosis, the value obtained in these two groups was different from the control group.

**Table 5:** ROC analysis results of nesfatin values in acute and control groups.

	Nesfatin
Cut-off point	≤30.62
AUC (%95 CI)	0.773 (0.673–0.874)
*p*	<0.001
Sensitivity	77.03
Specificity	83.33
PPV	90.48
NPV	63.83

Abbreviations: AUC: area under the curve, *p*: *p*-value, PPV: positive predictive value, NPV: positive predictive value. The place with the “Maximum Youden Index” value was taken as the most appropriate cut-off value.

## Data Availability

The datasets used and/or analyzed during the current study are available from the corresponding author upon reasonable request.

## References

[1] Feigin V.L., Forouzanfar M.H., Krishnamurthi R., Mensah G.A., Connor M., Bennett D.A., Moran A.E., Sacco R.L., Anderson L., Truelsen T. (2014). Global Burden of Diseases, Injuries, and Risk Factors Study 2010 (GBD 2010) and the GBD Stroke Experts Group. Global and regional burden of stroke during 1990–2010: Findings from the Global Burden of Disease Study 2010. Lancet.

[2] Zhu N., Shu H., Jiang W., Wang Y., Zhang S. (2020). Mean platelet volume and mean platelet volume/platelet count ratio in nonvalvular atrial fibrillation stroke and large artery atherosclerosis stroke. Medicine.

[3] Flaherty M.L., Kissela B., Khoury J.C., Alwell K., Moomaw C.J., Woo D., Khatri P., Ferioli S., Adeoye O., Broderick J.P. (2013). Carotid artery stenosis as a cause of stroke. Neuroepidemiology.

[4] Oh-I S., Shimizu H., Satoh T., Okada S., Adachi S., Inoue K., Eguchi H., Yamamoto M., Imaki T., Hashimoto K. (2006). Identification of nesfatin-1 as a satiety molecule in the hypothalamus. Nature.

[5] Xu D., Yu Y., Xu Y., Ge J. (2021). Plasma Nesfatin-1: Potential Predictor and Diagnostic Biomarker for Cognitive Dysfunction in T2DM Patient. Diabetes Metab. Syndr. Obes..

[6] Lawrence A.J., Jarrott B. (1996). Neurochemical modulation of cardiovascular control in the nucleus tractus solitarius. Prog. Neurobiol..

[7] Shimizu H., Oh-I S., Okada S., Mori M. (2009). Nesfatin-1: An overview and future clinical application. Endocr. J..

[8] Recinella L., Orlando G., Ferrante C., Chiavaroli A., Brunetti L., Leone S. (2020). Adipokines: New Potential Therapeutic Target for Obesity and Metabolic, Rheumatic, and Cardiovascular Diseases. Front. Physiol..

[9] Saeidi A., Haghighi M.M., Kolahdouzi S., Daraei A., Abderrahmane A.B., Essop M.F., Laher I., Hackney A.C., Zouhal H. (2021). The effects of physical activity on adipokines in individuals with overweight/obesity across the lifespan: A narrative review. Obes. Rev..

[10] Naseroleslami M., Sharifi M., Rakhshan K., Mokhtari B., Aboutaleb N. (2023). Nesfatin-1 attenuates injury in a rat model of myocardial infarction by targeting autophagy, inflammation, and apoptosis. Arch. Physiol. Biochem..

[11] Mimee A., Smith P.M., Ferguson A.V. (2012). Nesfatin-1 influences the excitability of neurons in the nucleus of the solitary tract and regulates cardiovascular function. Am. J. Physiol.-Regul. Integr. Comp. Physiol..

[12] Altas M., Uca A.U., Akdag T., Odabas F.O., Tokgoz O.S. (2022). Serum levels of irisin and nesfatin-1 in multiple sclerosis. Arq. Neuropsiquiatr..

[13] Price C.J., Samson W.K., Ferguson A.V. (2008). Nesfatin-1 inhibits NPY neurons in the arcuate nucleus. Brain Res..

[14] Aydin S., Dag E., Ozkan Y., Arslan O., Koc G., Bek S., Kirbas S., Kasikci T., Abasli D., Gokcil Z. (2011). Time-dependent changes in the serum levels of prolactin, nesfatin-1 and ghrelin as a marker of epileptic attacks young male patients. Peptides.

[15] Wei Y., Li J., Wang H., Wang G. (2018). NUCB2/nesfatin-1: Expression and functions in the regulation of emotion and stress. Prog. Neuropsychopharmacol. Biol. Psychiatry.

[16] Vas S., Ádori C., Könczöl K., Kátai Z., Pap D., Papp R.S., Bagdy G., Palkovits M., Tóth Z.E. (2013). Nesfatin-1/NUCB2 as a potential new element of sleep regulation in rats. PLoS ONE.

[17] Xu Y.Y., Ge J.F., Liang J., Cao Y., Shan F., Liu Y., Yan C.Y., Xia Q.R. (2018). Nesfatin-1 and cortisol: Potential novel diagnostic biomarkers in moderate and severe depressive disorder. Psychol. Res. Behav. Manag..

[18] Ge J.F., Xu Y.Y., Qin G., Peng Y.N., Zhang C.F., Liu X.R., Liang L.C., Wang Z.Z., Chen F.H. (2015). Depression-like Behavior Induced by Nesfatin-1 in Rats: Involvement of Increased Immune Activation and Imbalance of Synaptic Vesicle Proteins. Front. Neurosci..

[19] Ari M., Ozturk O.H., Bez Y., Oktar S., Erduran D. (2011). High plasma nesfatin-1 level in patients with major depressive disorder. Prog. Neuropsychopharmacol. Biol. Psychiatry.

[20] Gunay H., Tutuncu R., Aydin S., Dag E., Abasli D. (2012). Decreased plasma nesfatin-1 levels in patients with generalized anxiety disorder. Psychoneuroendocrinology.

[21] Xu D., Kong T., Shao Z., Liu M., Zhang R., Zhang S., Kong Q., Chen J., Cheng B., Wang C. (2021). Orexin-A alleviates astrocytic apoptosis and inflammation via inhibiting OX1R-mediated NF-κB and MAPK signaling pathways in cerebral ischemia/reperfusion injury. Biochim. Biophys. Acta Mol. Basis Dis..

[22] White B.C., Sullivan J.M., DeGracia D.J., O’Neil B.J., Neumar R.W., Grossman L.I., Rafols J.A., Krause G.S. (2000). Brain ischemia and reperfusion: Molecular mechanisms of neuronal injury. J. Neurol. Sci..

[23] Erfani S., Moghimi A., Aboutaleb N., Khaksari M. (2019). Protective Effects of Nucleobinding-2 After Cerebral Ischemia Via Modulating Bcl-2/Bax Ratio and Reducing Glial Fibrillary Acid Protein Expression. Basic Clin. Neurosci..

[24] Angelone T., Filice E., Pasqua T., Amodio N., Galluccio M., Montesanti G., Quintieri A.M., Cerra M.C. (2013). Nesfatin-1 as a novel cardiac peptide: Identification, functional characterization, and protection against ischemia/reperfusion injury. Cell. Mol. Life Sci..

[25] Ayada C., Toru Ü., Genç O., Akcılar R., Şahin S. (2015). Balanced oxidative status by nesfatin-1 in intestinal ischemia-reperfusion. Int. J. Clin. Exp. Med..

[26] Kazimierczak-Kabzińska A., Marek B., Borgiel-Marek H., Kajdaniuk D., Kos-Kudła B. (2020). Assessing the blood concentration of new adipocytokines in patients with ischaemic stroke. Endokrynol. Pol..

[27] Mohd A.B., Alabdallat Y., Mohd O.B., Ghannam R.A., Sawaqed S., Hasan H., Ellebedy M., Turkmani K., Al-Ezzi S. (2023). Medical and Surgical Management of Symptomatic and Asymptomatic Carotid Artery Stenosis: A Comprehensive Literature Review. Cureus.

[28] Meng Q., Lu Q., Zhang Z., Liu J., Lou Y., Wang Y., Liu J. (2021). Nesfatin-1 inhibits free fatty acid (FFA)-induced endothelial inflammation via Gfi1/NF-κB signaling. Biosci. Biotechnol. Biochem..

[29] Adams H.P., Bendixen B.H., Kappelle L.J., Biller J., Love B.B., Gordon D.L., Marsh E.E. (1993). Classification of subtype of acute ischemic stroke. Definitions for use in a multicenter clinical trial. TOAST. Trial of Org 10172 in Acute Stroke Treatment. Stroke.

[30] Khatri R., McKinney A.M., Swenson B., Janardhan V. (2012). Blood-brain barrier, reperfusion injury, and hemorrhagic transformation in acute ischemic stroke. Neurology.

[31] Rosário M., Fonseca A.C. (2023). Update on Biomarkers Associated with Large-Artery Atherosclerosis Stroke. Biomolecules.

[32] Jickling G.C., Sharp F.R. (2015). Biomarker panels in ischemic stroke. Stroke.

[33] Kamtchum-Tatuene J., Jickling G.C. (2019). Blood Biomarkers for Stroke Diagnosis and Management. Neuromol. Med..

[34] Dagonnier M., Donnan G.A., Davis S.M., Dewey H.M., Howells D.W. (2021). Acute Stroke Biomarkers: Are We There Yet?. Front. Neurol..

[35] Tekin T., Cicek B., Konyaligil N. (2019). Regulatory Peptide Nesfatin-1 and its Relationship with Metabolic Syndrome. Eurasian J. Med..

[36] Chinapayan S.M., Kuppusamy S., Yap N.Y., Perumal K., Gobe G., Rajandram R. (2022). Potential Value of Visfatin, Omentin-1, Nesfatin-1 and Apelin in Renal Cell Carcinoma (RCC): A Systematic Review and Meta-Analysis. Diagnostics.

[37] He S., He Y., Jin F., Liu Y. (2021). Correlation analysis of IGF-1, ZAG, nesfatin-1, HbA1c levels, and type 2 diabetes mellitus complicated with hypothyroidism. Medicine.

[38] Zhou S., Nao J. (2024). Nesfatin-1: A Biomarker and Potential Therapeutic Target in Neurological Disorders. Neurochem. Res..

[39] Dai H., Li X., He T., Wang Y., Wang Z., Wang S., Xing M., Sun W., Ding H. (2013). Decreased plasma nesfatin-1 levels in patients with acute myocardial infarction. Peptides.

